# VITAMIN A DEFICIENCY IN BRAZILIAN CHILDREN AND ASSOCIATED VARIABLES

**DOI:** 10.1590/1984-0462/;2018;36;2;00013

**Published:** 2018-03-23

**Authors:** Daniela Braga Lima, Lucas Petri Damiani, Elizabeth Fujimori

**Affiliations:** a Universidade Federal de Alfenas, Alfenas, MG, Brasil.; b Universidade de São Paulo, São Paulo, SP, Brasil.

**Keywords:** Vitamin A deficiency, Child health, Child nutrition, Nutrition, public health, Deficiência de vitamina A, Saúde da criança, Nutrição da criança, Nutrição em saúde pública

## Abstract

**Objective:**

To analyze the variables associated with vitamin A deficiency (VAD) in Brazilian children aged 6 to 59 months, considering a hierarchical model of determination.

**Methods:**

This is part of the National Survey on Demography and Health of Women and Children, held in 2006. Data analysis included 3,417 children aged from six to 59 months with retinol data. Vitamin A deficiency was defined as serum retinol <0.7 mol/L. Univariate and multiple Poisson regression analysis were performed, with significance level set at 5%, using a hierarchical model of determination that considered three conglomerates of variables: those linked to the structural processes of community (socioeconomic-demographic variables); to the immediate environment of the child (maternal variables, safety and food consumption); and individual features (biological characteristics of the child). Data were expressed in prevalence ratio (PR).

**Results:**

After adjustment for confounding variables, the following remained associated with VAD: living in the Southeast [PR=1,59; 95%CI 1,19-2,17] and Northeast [PR=1,56; 95%CI 1,16-2,15]; in urban area [RP=1,31; 95%CI 1,02-1,72]; and mother aged ≥36 years [RP=2,28; 95%CI 1,37-3,98], the consumption of meat at least once in the last seven days was a protective factor [PR=0,24; 95%CI 0,13-0,42].

**Conclusions:**

The main variables associated with VAD in the country are related to structural processes of society and to the immediate, but not individual, environment of the child.

## RESUMO

**Objetivo:**

Analisar variáveis associadas à deficiência de vitamina A (DVA) em crianças brasileiras de 6 a 59 meses de idade, considerando um modelo hierárquico de determinação.

**Métodos:**

Trata-se de um recorte da Pesquisa Nacional de Demografia e Saúde da Criança e da Mulher (PNDS), realizada em 2006. A análise dos dados incluiu 3.417 crianças de seis a 59 meses com dados de retinol. A DVA foi definida como retinol sérico <0,7 mmol/L. Realizaram-se análises univariada e ajustada por regressão múltipla de Poisson, com nível de significância de 5%, utilizando-se modelo hierárquico de determinação que considerou três blocos de variáveis: vinculadas aos processos estruturais da sociedade (variáveis socioeconômicas e demográficas); ao ambiente imediato da criança (variáveis maternas, de segurança e consumo alimentar); e individuais (características biológicas da criança). Os dados foram expressos em razão de prevalência (RP).

**Resultados:**

Após ajuste para variáveis de confusão, permaneceram associadas à DVA: residir no Sudeste [RP=1,59; IC95% 1,19-2,17] e no Nordeste [RP=1,56; IC95% 1,16-2,15]; em zona urbana [RP=1,31; IC95% 1,02-1,72]; ter mãe com idade ≥36 anos [RP=2,28; IC95% 1,37-3,98]; sendo proteção consumir carne pelo menos uma vez nos últimos sete dias [RP=0,24; IC95% 0,13-0,42].

**Conclusões:**

As principais variáveis associadas à DVA no país relacionam-se aos processos estruturais da sociedade e ao ambiente imediato da criança, e não aos individuais.

## INTRODUCTION

Vitamin A deficiency (VAD) stands out as an important nutritional problem, especially in middle- and lower-income countries, with more obvious consequences at life stages with higher nutritional demand such as early childhood. VAD in children is one of the most important causes of preventable blindness and a major contributor to morbidity and mortality from infections, which affect the poorest segments of the population.^1^ ^,^ ^2^ ^,^ ^3^

The overall prevalence of VAD in children under five years of age was estimated at 33% from 1995 to 2005, a number that stands for a serious public health issue in 73 countries (prevalence >20%) and moderate (prevalence 10-20%) in 49 countries, including Brazil, where the prevalence was estimated at 13%.^4^ Reviews of Brazilian literature, however, have shown a median prevalence of 32%, quite above the estimates by the World Health Organization (WHO), especially in the regions of Vale do Jequitinhonha and Mucuri in the State of Minas Gerais, and Ribeira in the State of São Paulo.^5^ ^,^ ^6^ ^,^ ^7^

For the first time in the country, data on serum retinol were obtained in the last National Demography and Health Survey on Children and Women (PNDS in the Portuguese acronym), conducted in 2006, which revealed inadequate levels of vitamin A in 17.4% of children aged 6 to 59 months of age, with marked regional differences and persisting as a moderate public health problem in the country.^8^

Inadequate intake of dietary sources of vitamin A to meet physiological needs stands out as the main cause of VAD,^4^ but other variables have also been associated with childhood VAD, including social, economic, and environmental conditions,^3^ ^,^ ^9^ ^,^ ^10^ ^,^ ^11^ ^,^ ^12^ maternal characteristics,^9^ ^,^ ^13^ ^,^ ^14^ nutritional status,^6^ infections^13^ ^,^ ^14^ ^,^ ^15^, and children’s age range.^9^ ^,^ ^16^ However, these associations are not always considered, since the studies are not conclusive in identifying factors associated with infant VAD.

Aiming to contribute to the understanding of variables associated with VAD in the field of collective health, which conceives health-disease dyad as a socially determined process,^17^ the present study assumes that what determines VAD is an interrelation of elements of different dimensions that should be studied through a hierarchical approach. A study carried out in Pernambuco, which adopted a hierarchical explanatory model, pointed several aspects still lacking clarification so one can understand the variables associated with VAD.^10^ ^,^ ^13^

Thus, even being acknowledged as a relevant and well-exploited problem, there are still epidemiological spectra to be investigated, with emphasis to the fact that, in this context, few studies have analyzed food safety and consumption related to children. Despite being disclosed in a public report, the data about serum retinol first nationally obtained by PNDS/2006 were not explored with respect to these variables, so we considered pertinent to carry out this analysis in Brazilian children, on the basis of a hierarchical model - purpose of this paper.

## METHOD

This is a cut from PNDS/2006, whose data are in public domain and available online (http://bvsms.saude.gov.br/bvs/pnds/banco_dados.php).

PNDS/2006 was approved by a national ethics committee and conducted in accordance with ethical standards.^8^ It is a cross-sectional study of national representativeness whose purpose was to characterize the population of women of child-bearing age and children under five years of age in the five macro-regions of the country. The description of the method, including sampling and selection techniques in multi-stage analysis units, procedures for data collection, internal consistency checking, laboratory analysis, anthropometric measurement means, and ethical aspects are available in the official PNDS 2006 report. Serum retinol levels have been determined in 3,499 children.^8^

The present study analyzed data from 3,417 children, 82 being excluded because of blood samples unsuitable for retinol analysis. To evaluate the accuracy and validity of the sample, the design effect (Deff) was calculated and the sample weights adopted by PNDS/2006 were considered, resulting in a large sample of 9,206,000 children.

Serum retinol levels were assessed by high performance liquid chromatography (HPLC).^8^ The criteria recommended by WHO were used to classify VAD as to level of epidemiological importance for public health:^18^ mild importance for prevalence <10%; moderate for 10 to 20%; and severe for >20%.

**Figure 1:**
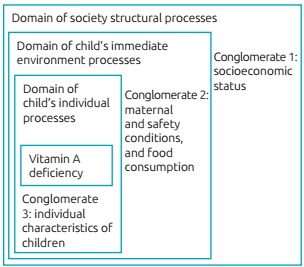
Hierarchical model proposed for the analysis of variables associated with vitamin A deficiency.

Source: Adapted from Oliveira et al.^10^ and Miglioli et al.^13^.

VAD was the outcome variable. The exploratory variables were categorized into three conglomerates, while sticking to a hierarchical model for VAD determination previously elaborated based on the theoretical relations proposed to explain its occurrence.^10^ ^,^ ^13^ The first one (conglomerate 1) held variables relating to structural processes, determined by social and economic policies that directly affect the population’s living conditions (socioeconomic and demographic conditions). The second one (conglomerate 2) encompassed variables related to the immediate environment the child was part of (maternal variables, food safety and consumption); and the last one (conglomerate 3) was consisted of infant variables (children’s individual characteristics) (Figure 1).

Exploratory variables of conglomerate 1: macroregion of residence (North, Northeast, Southeast, South, and Midwest); area of residence (urban and rural); socioeconomic status (A to E) according to the Brazilian Association of Research Companies^19^, and per capita income (<0.5 minimum wage and ≥0.5 minimum wage). In conglomerate 2, maternal variables analyzed were: age (<20 years, 20 to 35 years, and ≥36 years), and years of study (0 to 4 years, 5 to 8 years, and 9 and over); food safety, as per the Brazilian Scale of Food Insecurity (composed of 15 questions assessing their concern with lack of food, impairment of family food quality, and quantitative restriction on food availability, with families classified according to the following four categories: food safety, mild unsafety, moderate unsafety, severe unsafety);^20^ and food consumption over the last seven days, according to food groups (Cereals/dough and pasta, vegetables, fruits, beans, sweets).

The exploratory variables of block 3 included infant characteristics: age (<2 years and ≥2 years); gender (female or male); breastfeeding at any time (yes/no); exclusive breastfeeding (EBF) (<30 days and ≥30 days); Total time of breastfeeding (BF) (<6 months, 6 to 11 months, ≥12 months); supplementation of vitamin A and iron (yes/no); hospitalization over the last 12 months (yes/no). Anemia (yes/no) was defined as hemoglobin (Hb) <11 g/dL, assessed by the cyanometahemoglobin method;^8^ nutritional status was analyzed by body mass index per age (BMI/age), according to WHO reference standard for Z score, as eutrophic (-2 Z score ≤BMI/age ≤+2 Z score), lean (BMI <-2 Z score), and overweight (BMI >+2 Z score).^21^

The parameters and respective 95% confidence intervals (95%CI) were estimated for the expanded data while considering the sample design effect. The strength of association between the outcome variable and the exploratory variables was analyzed by prevalence ratio (PR) and Poisson regression, with a significance level at 5%.

Upon the multiple analysis, the input of exploratory variables to the model followed a previously established hierarchical order. Variables were picked by stepwise forward selection for the model, the value p≥0.20 being the exclusion criterion at each step. However, the variables economic level; food safety; maternal years of study; consumption of vegetables, fruits and beans; age; hospitalization in previous 12 months; and BMI/age of children not presenting significance at p<0.20 level in the univariate analysis were maintained in the final model of VAD, in view of their relevance when determining this deficiency.^10^ ^,^ ^13^

The statistical package “R” version 2.12.2 (R Foundation for Statistical Computing, Vienna, Austria, http://www.R-project.org) was used for the analysis.

## RESULTS

VAD prevalence was 17.5% (95%CI 15.1-20.2%) and Table 1 shows the results of crude analyses for the studied outcome. In the univariate analysis, the variables of conglomerate 1 and part of conglomerate 2 statistically associated (p <0.05) with the occurrence of VAD were: macroregion of residence (p<0.001), with higher incidence in the Southeast and Northeast; residence in urban area (p=0.027), and maternal age above 36 years (p<0.001). The variable maternal years of study was not significantly associated with VAD; however, children of mothers with less than five years of schooling were pointed as more likely to have VAD. Added to that, a significant number of children with AVD were found to be in severe food unsafety, but no significant association between the variables analyzed was found.

**Table 1:** Prevalence of Vitamin A deficiency in children aged 6 to 59 months according to socioeconomic, mother, and food safety variables.

	Children with available data	Prevalence of retinol (<0.7 µmol/L) vitamin A deficiency (%)	PR	95%CI	p-value
Conglomerate 1					
Macroregion					
Midwest	667	12.1	1.000		<0.001
Northeast	657	19.3	1.590	1.13-2.23	
North	822	9.8	0.810	0.55-1.18	
Southeast	672	22.1	1.820	1.28-2.59	
South	599	10.1	0.830	0.56-1.25	
Region of residence					
Rural	1.268	13.3	1.000		0.027
Urban	2.149	18.7	1.410	1.04-1.91	
Family socioeconomic status					
A	26	29.0	1.000		0.844
B	374	18.3	0.630	0.23-1.72	
C	1.600	17.8	0.610	0.26-1.46	
D	877	17.3	0.600	0.25-1.45	
E	539	15.2	0.520	0.21-1.31	
Per capita income (minimum wage)					
<0.5	1.889	16.2	0.790	0.57-1.10	0.167
≥0.5	925	20.4	1.000		
Mother’s years of study					
0-4	1.476	16.8	1.040	0.75-1.43	0.406
5-8	1.021	20.2	1.250	0.86-1.81	
9 and older	823	16.2	1.000		
**Conglomerate 2**					
Mother’s age (years old)					
15-20	262	11.7	1.000		<0.001
20-36	2.716	15.9	1.360	0.80-2.29	
36-50	439	31.4	2.680	1.47-4.90	
Food safety					
Food safety	1.556	16.8	1.000		0.317
Mild unsafety	913	19.1	1.137	0.81-1.60	
Moderate unsafety	498	14.0	0.834	0.55-1.27	
Severe unsafety	344	22.6	1.342	0.89-2.02	

PR: prevalence ratio; 95%CI: 95% confidence interval.

As to food consumption in the last seven days (conglomerate 2), the prevalence of VAD was statistically associated (p<0.05) with meat consumption as a protective factor (PR=0.60, 95%CI 0.41-0.88). The food basis of children was found to be represented by the group of cereals/dough and pasta (n=2,630) and beans (n=2,193), for these were consumed “every day”, with inadequacy in other food groups’ consumption (Table 2).

**Table 2:** Prevalence of vitamin A deficiency in children aged 6 to 59 months according to the food consumption and food groups in the previous seven days.

	Children with available data	Prevalence of retinol (<0.7 µmol/L) vitamin A deficiency (%)	PR	95%CI	p-value
Conglomerate 2					
Cereals/dough and pasta (days)					
Did not eat	113	23.5	1		0.899
1	99	21.0	0.9	0.34-2.40	
2-3	326	17.7	0.75	0.40-1.40	
4-6	223	18.5	0.79	0.28-2.25	
Every day	2.630	17.2	0.73	0.43-1.23	
Vegetables (days)					
Did not eat	1.035	17.7	1		0.753
1	354	16.0	0.905	0.56-1.46	
2-3	885	18.1	1.026	0.67-1.57	
4-6	472	14.7	0.831	0.53-13.1	
Every day	637	20.1	1.141	0.80-1.63	
Fruit (days)					
Did not eat	503	14.0	1		0.405
1	289	20.1	1.44	0.85-2.44	
2-3	844	16.3	1.17	0.74-1.85	
4-6	510	15.1	1.08	0.65-1.78	
Every day	1.242	19.5	1.4	0.91-2.14	
Beans (days)					
Did not eat	311	15.1	1		0.249
1	171	19.5	1.29	0.65-2.56	
2-3	435	14.7	0.97	0.57-1.67	
4-6	271	11.8	0.78	0.41-1.50	
Every day	2.193	19.1	1.26	0.79-2.03	
Types of meat (days)					
Did not eat	608	25.1	1		<0.001
1	326	6.8	0.27	0.15-0.50	
2-3	994	21.6	0.86	0.60-1.24	
4-6	572	13.2	0.53	0.34-0.81	
Every day	881	15.0	0.6	0.41-0.88	
Dairy (days)					
Did not eat	1.165	16.6	1		0.278
1	393	18.5	1.12	0.71-1.77	
2-3	741	14.0	0.85	0.57-1.25	
4-6	466	18.2	1.1	0.74-1.64	
Every day	619	21.8	1.31	0.88-1.97	
Sweets (days)					
Did not eat	1.042	18.4	1		0.154
1	490	14.8	0.8	0.54-1.21	
2-3	728	22.0	1.19	0.82-1.74	
4-6	453	11.8	0.64	0.41-1.01	
Every day	658	17.7	0.96	0.65-1.43	

PR: prevalence ratio; 95%CI: 95% confidence interval.

No statistical association was seen between VAD and individual characteristics of children in the univariate analysis (conglomerate 3) (Table 3). The high incidence of VAD (16.9%) among children who used prophylactic vitamin A supplementation (not shown in table) drew attention.

**Table 3:** Prevalence of Vitamin A deficiency in children aged 6 to 59 months according to infant variables.

	Children with available data	Prevalence of retinol (<0.7 µmol/L) vitamin A deficiency (%)	PR	95%CI	p-value
Conglomerate 3					
Age (years)					
<2	1.070	16.9	1.00		0.706
≥2	2.347	17.8	1.06	0.80-1.40	
Gender					
Female	1.627	16.4	1.00		0.418
Male	1.790	18.5	1.13	0.84-1.51	
Breastfed					
No	118	25.5	1.00		0.140
Yes	3.295	17.2	0.68	0.41-1.12	
EBF time (days)					
<30	689	15.1	1.00		0.387
≥30	2.529	17.7	1.17	0.82-1.68	
BF time (months)					
< 6	862	16.6	1.00		0.689
6-11	638	14.2	0.86	0.57-1.29	
12 or more	1.036	17.2	1.04	0.69-1.55	
Hospitalization in 12 previous months					
No	2.994	17.3	1.00		0.581
Yes	422	19.1	1.11	0.78-1.57	
Nutritional status (BMI/age)					
Eutrophy	3.033	17.5	1.00		0.626
Underweight	64	12.5	0.72	0.27-1.91	
Overweight	249	20.1	1.15	0.76-1.74	
Anemia (hemoglobin)					
No (≥11 g/dL)	2.861	17.1	1.00		0.480
Yes (<11 g/dL)	556	19.2	0.89	0.65-1.22	

PR: prevalence ratio; 95%CI: 95% confidence interval; EBF: exclusive breastfeeding; BF: breastfeeding; BMI: body mass index.

Table 4 shows the variables that remained in the multiple analysis model after adjustment. From conglomerate 1, AVD was associated with: residence in the northeast (PR=1.56, 95%CI 1.16-2.15) and southeast macroregions (PR=1.59, 95%CI, 1.19-2.17); in the urban area (PR=1.31, 95%CI 1.02-1.72); mother aged ≥36 years (PR 2.28, 95%CI 1.37-3.98), with meat consumption at least once in the last seven days being a protective factor (PR=0.24, 95%CI, 0.13-0.42) (conglomerate 2).

**Table 4:** Final multiple model for variables associated with Vitamin A deficiency.

	Prevalência de deficiência de vitamina A (%) retinol (<0.7 µmol/L)	RP AJ	IC95%	p-value	Deff*
Conglomerate 1					
Macroregion					
Midwest	12.1	1.00		<0.001	1.57
Northeast	19.3	1.56	1.16-2.15		
North	9.8	0.77	0.54-1.10		
Southeast	22.1	1.59	1.19-2.17		
South	10.1	0.78	0.54-1.14		
Region of residence					
Rural	13.3	1.00		0.042	1.66
Urban	18.7	1.31	1.02-1.72		
**Conglomerate 2**					
Mother’s age (years old)					
15-20	11.7	1.00		0.002	2.37
20-36	15.9	1.36	0.86-2.31		
36-50	31.4	2.28	1.37-3.98		
Meat ingestion (days)					
Did not eat	25.1	1.00		<0.001	2.39
1	6.8	0.24	0.13-0.42		
2-3	21.6	0.77	0.58-1.04		
4-6	13.2	0.55	0.37-0.80		
Every day	15.0	0.61	0.43-0.86		

Ad PR: adjusted prevalence ratio; 95%CI: 95% confidence interval; Deff: design effect.

## DISCUSSION

In the context of variables related to structural processes, VAD was associated with the macroregion of residence, with higher prevalence among children living in the southeast, one of the most developed regions of Brazil, similarly to children living in the northeast, one of the poorest regions of the country. This shows that VAD is not restricted to microregions presenting the greatest severity of VAD disorders, such as the Vale do Jequitinhonha and Mucuri regions in the State of Minas Gerais, and Ribeira in the State of São Paulo.^5^ ^,^ ^6^

Thus, one could questioned whether VAD would have a trans-social character, once it affects both the least and the most developed macroregions of the country and whether the National Vitamin A Supplementation Program (Vitamina A Mais) should be expanded, once its expansion has contemplated all municipalities in the northeast region since the 1980s; the ones composing the Legal Amazon since 2010 and, since 2012, all municipalities in the north, 585 municipalities member of the program “*Brasil Sem Miséria*” (Brazil without Extreme Poverty) in the midwest, south and southeast regions, as well as all indigenous special health districts.^7^

Although the population living in rural areas is the most vulnerable to nutritional deficiencies worldwide, as they face greater difficulty in accessing health services, education, and acquiring food of better nutritional value,^4^ in Brazil, VAD prevails in urban areas, as verified in the State of Pernambuco,^13^ but contrary to what happened in the semi-arid region of the State of Alagoas.^22^ The intense urbanization process that the country has experienced in the last decades could justify this result, from 56% in 1970 to 84% in 2010, as well as the increasing metropolitan agglomerations in absolute terms from 27 to 70 million between 1970 and 2010.^23^ This change favors economic activities, but also diffuses new patterns of social relations and life styles that exacerbate inequality, resulting in people living in precarious conditions, especially in the periphery, with important impact on living conditions due to the lack of work opportunities, low wages, and unhealthy housing conditions, all negatively interfering with people’s health status.^23^

Moreover, compared to the urban area residents, people from the rural area consume more basic and better-quality food, predominantly rice, beans, cassava, sweet potatoes, fruits, meat, pork, chicken, and fish, while ultra-processed food intake predominate in urban areas.^24^ ^,^ ^25^ ^,^ ^26^ Thus, the locus of poverty and child malnutrition appears to have gradually changed from rural to urban areas. It is noteworthy that even though these children seem to be well fed, with enough calories to maintain their daily activities, they may suffer from “hidden hunger”, caused by the lack of micronutrients such as vitamin A, iron or zinc, all essential for child growth and development as herein stated.

When it comes to structural processes, there was no evidence of association between VAD and social class or per capita income. This result has also been obtained in national and international population studies.^3^ ^,^ ^10^ ^,^ ^11^ ^,^ ^12^ ^,^ ^13^ ^,^ ^15^ In fact, with the exception of extreme poverty situations, income does not seem to act as a factor associated with this deficiency, reinforcing the thesis that inadequate intake of food containing this micronutrient could be the main cause of VAD.^4^ ^,^ ^24^

The matter of food safety and VAD has been little investigated in Brazil. Only two studies conducted in the Northeast are highlighted, one of which showed lower retinol levels among children from families considered to live in moderate to severe food unsafety, but with no statistical association,^10^ and the other did not find any association,^27^ similarly to the present study. When it comes to the child’s immediate environment, association between VAD and low maternal schooling, often found in national and international surveys, is also emphasized,^6^ ^,^ ^9^ ^,^ ^11^ ^,^ ^14^ but was not pointe out in Pernambuco.^13^ Not finding any association between this micronutrient deficiency and important structural variables or children’s immediate environment calls for deeper inquiry, but this result suggests that VAD is likely to derive from poor dietary sources of vitamin A such as products of animal origin or containing beta-carotene, because feeding habits, besides being dependent of economic conditions, is a cultural practice.^11^

Thus, this analysis of children’s food consumption at the national level fills an important gap in the investigations about variables associated with VAD in the country. In fact, the consumption of meat once in the last seven days remained, in the final model, as a protective against the nutritional deficiency studied, adding importance to the role of diet, when it comes to vitamin A consumption and bioavailability, as the main factor associated with VAD.^4^ ^,^ ^24^ This derives from the greater bioconversion (absorption + bioavailability) of vitamin A present in products of animal origin (retinol) compared to the form it is found in plant-origin products (carotenoids with provitamin A activity).^7^ ^,^ ^24^ Thus, the consumption of meat, a source of preformed vitamin A, is a complementary data that reinforces the explanatory model of serum retinol levels found in the children of our sample.

In addition, it was reiterated that children’s feeding basis was represented by the group of cereals/dough/pasta and beans, consumed “every day”, while almost one third of other food groups were not consumed, including dairy products (even children aged 6 to 59 months), vegetables; low consumption of fruit, important sources of vitamin A, in the week prior to study was also noted. Such feeding pattern had already been detected in the 1990s and, along with breastfeeding interruption and early introduction of complementary food, explained the greater incidence of VAD in children younger than 24 months.^28^ Despite the evidence that children between 12 and 48 months of age have lower consumption of vitamin-A rich food compared to the other age groups,^24^ this study found no difference as to the prevalence of VAD according to age.

VAD was associated with maternal older age, contradicting a previous study which explained higher frequency of VAD in urban children of younger mothers with maternal inexperience for the care of their children, resulting in insufficient provision of vitamin A.^13^ The greater participation of women in the labor market could partially explain this finding, although a study with children from urban areas of nine municipalities in the State of Paraíba, northeast region of Brazil, has not pointed out any association between VAD and maternal age.^15^

This analysis did not find associations with individual variables of the child, which suggests that VAD, like other privation-related problems, is tied to structural processes of society and to the immediate environment of children and lingers in low-income countries and continents, as well as in less favored regions and families.^11^ Thus, the incidence found in the southeast region, the richest macroregion of the country, requires more detailed investigation for deep understanding.

It should be noted that, even with the decrease found, the prevalence of childhood VAD in Brazil remains higher than WHO’s estimate (13.3%) and a moderate public health problem in the country.^4^ However, not having infectious processes evaluated is an important limitation of this study, as they tend to decrease serum retinol concentrations in the first 24 hours of installation,^29^ which means that the prevalence may have been overestimated. Having food consumption in the last seven days assessed, on the other hand, is a substantial step forward in studies of this nature.

One could hypothesize that VAD reduction is related to prevention and control strategies adopted by the government, such as the interventions proposed under the National Food and Nutrition Policy and the National Vitamin A Supplementation Program, which recommends the distribution of vitamin A megadose capsules to children aged 6 to 59 months in areas considered to be at risk for this nutritional deficiency.^7^ ^,^ ^25^ Results≈indicate, however, that VAD control must be extended beyond areas at risk, as there is evidence that vitamin A supplementation reduces the mortality of children aged 6 to 59 months by 24%.^2^ However, it is worth noting that VAD was shown high even among children under vitamin A supplementation (16.9%), which may be related to the use of medication to treat VAD already installed, but not as prophylaxis, as recommended; this further reinforces the need for improvements in operations of the National Vitamin A Supplementation Program, with view to both preventing and controlling this deficiency.

This study investigated data dated back to 11 years ago, but it is emphasized that, up to the present time, the PNDS/2006 is the only source of VAD data available to the national level about Brazilian children, and this study used complex sampling technique to guarantee data legitimacy. The hierarchical analysis of variables associated with childhood VAD cross the country has proved especially important for the review of policies and the establishment of interventions that can reduce damages caused by this deficiency in the child population. However, as the cross-sectional design of the study does not allow causal assumptions, the findings should be interpreted with caution.

In summary, the results indicate that the main variables associated with VAD across the country are related to structural processes of society and the immediate, but not individual, environment of the child. Thus, controlling this nutritional deficiency that persists as a moderate public health problem requires investments not only in the healthcare field. In addition to starting the supplementation program where it does not exist yet, like the southeast region, and strengthening the coverage where it is weak, one must seek more sustainable solutions such as improving the intake of vitamin-a rich food.
